# The mechanisms by which polyamines accelerate tumor spread

**DOI:** 10.1186/1756-9966-30-95

**Published:** 2011-10-11

**Authors:** Kuniyasu Soda

**Affiliations:** 1 Department of Surgery and Cardiovascular Research Institute, Saitama Medical Center, Jichi Medical University, 1-847 Amanuma, Omiya, Saitama-city, Saitama (330-0834), Japan

**Keywords:** Polyamine, metastasis, spermine, spermidine, LAK, LFA-1

## Abstract

Increased polyamine concentrations in the blood and urine of cancer patients reflect the enhanced levels of polyamine synthesis in cancer tissues arising from increased activity of enzymes responsible for polyamine synthesis. In addition to their *de novo *polyamine synthesis, cells can take up polyamines from extracellular sources, such as cancer tissues, food, and intestinal microbiota. Because polyamines are indispensable for cell growth, increased polyamine availability enhances cell growth. However, the malignant potential of cancer is determined by its capability to invade to surrounding tissues and metastasize to distant organs. The mechanisms by which increased polyamine levels enhance the malignant potential of cancer cells and decrease anti-tumor immunity are reviewed. Cancer cells with a greater capability to synthesize polyamines are associated with increased production of proteinases, such as serine proteinase, matrix metalloproteinases, cathepsins, and plasminogen activator, which can degrade surrounding tissues. Although cancer tissues produce vascular growth factors, their deregulated growth induces hypoxia, which in turn enhances polyamine uptake by cancer cells to further augment cell migration and suppress CD44 expression. Increased polyamine uptake by immune cells also results in reduced cytokine production needed for anti-tumor activities and decreases expression of adhesion molecules involved in anti-tumor immunity, such as CD11a and CD56. Immune cells in an environment with increased polyamine levels lose anti-tumor immune functions, such as lymphokine activated killer activities. Recent investigations revealed that increased polyamine availability enhances the capability of cancer cells to invade and metastasize to new tissues while diminishing immune cells' anti-tumor immune functions.

## 1. Introduction

Polyamines, which include spermidine and spermine, are polycations with three or four amine groups. Almost all cells can produce polyamines, but their production is especially high in rapidly growing cells. Polyamine concentrations are often increased in the blood and urine of cancer patients, and these increased levels have been shown to correlate with poor prognosis [1]. The increased blood and urinary polyamine levels are attributable to increased polyamine synthesis by cancer cells, since these increases can be abolished by complete eradication of tumors by surgery or radio-chemotherapy [2-5]. The capacity of cancer tissue to produce abundant polyamines likely contributes to cancer cells' enhanced growth rates because polyamines are indispensable for cellular growth, which may at least partially explain why cancer patients with increased polyamine levels have a poorer prognosis [4-9]. However, an important factor that determines the malignant potential of cancer cells is the capability of cells to invade to surrounding tissues and to metastasize to distant organs. Therefore, it is important to understand the role of polyamines in cancer invasion and metastasis. In this review, recent experimental results from our and other groups are discussed.

## 2. What are polyamines?

**Figure 1:**
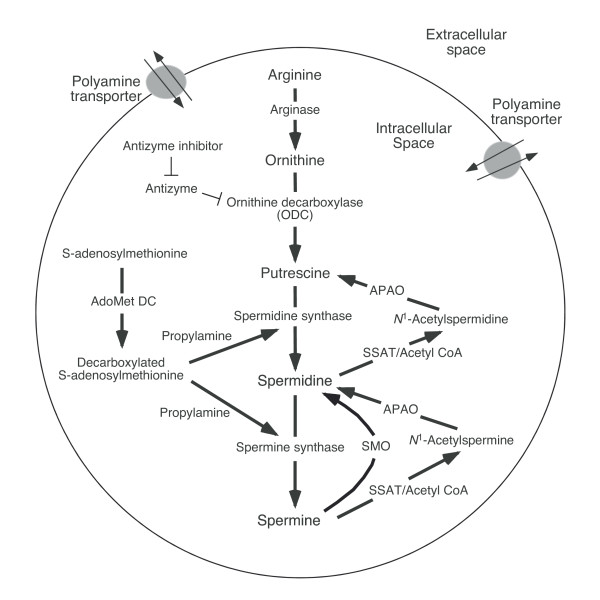
**Polyamine biosynthesis, degradation, and transmembrane transport**. The polyamines spermine and spermidine are synthesized from arginine. Arginase converts arginine to ornithine, and ornithine decarboxylase (ODC) catalyzes decarboxylation of ornithine to form putrescine, a polyamine precursor containing two amine groups. ODC, a rate-limiting enzyme with a short half-life, is inhibited by antizyme, and antizyme is inhibited by an antizyme inhibitor. S-adenosylmethionine decarboxylase (AdoMetDC) is the second rate-limiting enzyme in polyamine synthesis and is involved in the decarboxylation of S-adenosylmethionine. Spermidine synthetase and spermine synthase are constitutively expressed aminopropyltransferases that catalyze the transfer of the aminopropyl group from decarboxylated S-adenosylmethionine to putrescine and spermidine to form spermidine and spermine, respectively. Polyamine degradation is achieved by spermine/spermidine N^1^-acetyltransferase (SSAT) and N^1^-acetylpolyamine oxidase (APAO). In addition, spermine oxidase (SMO) specifically oxidizes spermine. Polyamines are transported across the membrane transmembrane by the polyamine transporter.

The natural polyamines, spermidine, and spermine, are found in almost every living cell at high micromolar to low millimolar quantities [10]. Polyamines are synthesized from arginine and s-adenosylmethionine with arginase converting arginine to ornithine, and ornithine decarboxylase (ODC) catalyzing ornithine decarboxylation to form putrescine, a polyamine precursor containing two amine groups (Figure 1). Polyamines are involved in diverse functions involved in cell growth and differentiation, such as DNA synthesis and stability, regulation of transcription, ion channel regulation, and protein phosphorylation [11-14].

Intracellular spermine and spermidine are degraded by spermidine/spermine N^1^-acetyltransferase (SSAT) and N^1^-acetylpolyamine oxidase (APAO). SSAT, a highly inducible enzyme, catalyzes the transfer of an acetyl group from acetyl-coenzyme A to the aminopropyl moiety of spermine and spermidine. APAO was previously described as polyamine oxidase but it preferentially catalyzes the oxidation of the *N*^1^-acetylspermine and *N*^1^-acetylspermidine produced by SSAT activity. This oxidation results in the production of H_2_O_2_, 3-acetoaminopropanal, and putrescine or spermidine (Spd), depending on the initial substrate [15-17]. Mammalian spermine oxidase (SMO) is an inducible enzyme that specifically oxidizes spermine, with the production of H_2_O_2_, 3-aminopropanal (3AP) and spermidine [16,17].

In addition to *de novo *synthesis and degradation, cellular polyamine concentrations are also regulated by transmembrane transport where cells take up polyamines from their surroundings or export them to the extracellular space (Figure 1).

## 3. Polyamines and cancer

Polyamine biosynthesis is up-regulated in actively growing cells, including cancer cells [10,18,19], therefore polyamine concentration as well as gene expression and activity of enzymes involved in polyamine biosynthesis, especially ODC, are higher in cancer tissues than in normal surrounding tissues [8,20-25].

Numerous reports have shown that both blood and urine polyamine concentrations are often increased in cancer patients [4,5,7,8,10]. A close correlation between blood polyamine levels and the amount of urinary polyamines has also been found in cancer patients [1]. Moreover, these levels decrease after tumor eradication and increase after relapse [2-5,23], indicating that polyamines synthesized by cancer tissues are transferred to the blood circulation and kidney, where they are excreted into the urine [26].

Polyamines are also produced in other parts of the body and can be transported to various organs and tissues such as the intestinal lumen where polyamines are absorbed quickly to increase portal vein polyamine concentrations [27]. The majority of spermine and spermidine in the intestinal lumen is absorbed in their original forms because there is no apparent enzymatic activity present to catalyze their degradation [28]. Polyamines absorbed by the intestinal lumen are distributed to almost all organs and tissues in the body [29] as demonstrated by the increased blood polyamine levels in animals and humans produced in response to continuous enhanced polyamine intake for six and two months, respectively [30,31]. However, short-term increased polyamine intake failed to produce such increases [30-32], possibly because of the homeostasis that inhibits acute changes in intracellular polyamine concentration. On the other hand, reductions in blood polyamine concentration were not achieved only by restricting oral polyamine intake. As such, at least two sources of intestinal polyamines are postulated: foods and intestinal microbiota. Decrease in blood polyamine levels can be successfully achieved by eliminating intestinal microbiota in addition to restricting food polyamines [33]. Taken together, these results indicate that polyamines are not only produced by cancer tissues but are also supplied from the intestinal lumen and together appear to influence polyamine levels in the body of cancer patients.

### 3. Polyamines in the body

In vitro experiments showed that cultured cells take up polyamines from their surroundings [34,35]. In blood circulation, the majority of polyamines are contained in blood cells, especially in red and white blood cells, and therefore increases in blood polyamine concentration indicate concurrent increases in polyamine levels in blood cells [36]. Similarly, intracellular polyamine concentrations in cells of otherwise normal tissues and organs in cancer patients can be increased [37]. One examination showed that spermidine and spermine levels are increased in the normal colon mucosa of cancer patients compared to the normal colon mucosa from patients without cancer [37], although another study was unable to detect these differences [38]. Given that polyamine concentrations are increased in the blood cells of cancer patients and numerous blood cells with increased polyamine concentrations exist in normal tissues, the polyamine concentration in normal tissues of cancer patients with increased blood polyamine levels might also be increased. In addition, orally administered radiolabeled polyamines have been shown to be immediately distributed to almost all organs and tissues [29,39,40].

Polyamine concentrations in the blood vary considerably among healthy individuals such that concentrations are not necessarily higher in cancer patients than in otherwise normal subjects [41,42] and this wide variation precludes the use of polyamine levels as a tumor marker as well as making detection of differences in polyamine concentrations in normal tissues of cancer patients and normal subjects difficult. The kinesis of polyamines may allow distant tissues and organs to influence polyamine levels of all cells in an organism.

## 4. Polyamines and cancer spread

Patients with increased polyamine levels either in the blood or urine are reported to have more advanced disease and worse prognosis compared to those with low levels, regardless of the type of malignancy [4-9]. Because polyamines are essential for cell growth, the increased capability of polyamine synthesis could reflect enhanced tumor proliferation. Therefore, inhibition of polyamine synthesis and availability by cancer cells could retard cancer cell growth. The efficacy of polyamine depletion is prominent in animal experiments. Inhibition of polyamine synthesis by DL-α-difluoromethylornithine (DFMO), an inhibitor of ODC that catalyzes the first rate-limiting step in polyamine biosynthesis, with or without methylglyoxal-bis-guanylhydrazone (MGBG), an inhibitor of S-Adenosylmethionine (SAM) that is required for polyamine synthesis, successfully suppressed tumor growth and prolonged survival of tumor-bearing animals [43-46]. Although the efficacy of polyamine restriction is not as apparent in humans as in animals [47,48], inhibition of polyamine synthesis by DFMO successfully suppressed the progression of neoplastic disease [49-52].

However, a major factor that directly influences the prognosis of patients with malignant disease is the capability of cancer cells to invade surrounding tissues and organs and evade immune cell defenses to metastasize to distant organs. In animal experiments, inhibition of polyamine synthesis by DFMO and/or MGBG not only reduced tumor growth but also decreased the amount of metastasis, resulting in prolonged survival of tumor bearing animals [43,44,46,53-55]. Therefore, the effect of polyamines on the metastatic potential of cancer cells, the host's anti-tumor immunity, and the corresponding mechanisms involved should be taken into consideration.

## 5. Mechanism of metastasis and involvement of polyamines (Figure 2)

There are several steps that occur during metastasis: separation of cancer cells from the tumor cluster (5-a); transmigration of cells from the original cluster to the circulation (5-b); and rooting and colonization in new organs and tissues (5-c) [56,57]. In addition, metastasis is completed only when cancer cells can successfully escape from the anti-tumor immune function of the host during this process (5-d). In this section, the mechanism of cancer metastasis and the involvement of polyamines are discussed.

### 5-a. Separation of cancer cells from the tumor cluster, and the role of polyamines

Cancer metastasis begins when cancer cells separate from the tumor cluster. This separation is initiated by decreased cell adhesion, which is normally maintained by the presence of adhesion molecules involved in intercellular binding and binding between cells and the extracellular matrix. Hypoxia, a common condition in cancer tissues, exerts a strong pressure on cells to separate from the tumor cluster and migrate into circulation [58,59]. Despite their *de novo *angiogenesis, solid tumors have scattered regions where oxygen delivery is compromised due to diffusion limitations, structural abnormalities of tumor microvessels, and disturbed microcirculation [60]. The cellular response to hypoxia involves the stabilization and resultant increase in levels of hypoxia inducible factor-1 (HIF-1), a transcription factor that enhances gene expression to promote angiogenesis, anaerobic metabolism, cell survival, and invasion [61]. Among these, suppression of adhesion molecules induced by hypoxia-induced HIF-1 stabilization is a strong selective pressure that enhances outgrowth of cells with high-grade malignancy. CD44 and E-cadherin are adhesion molecules whose expression decreases in response to hypoxia [62,63].

**Figure 2:**
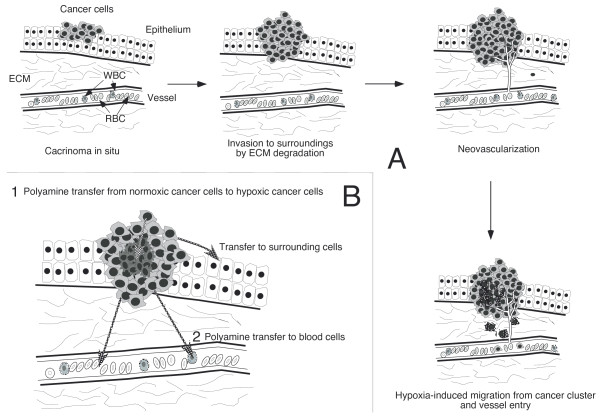
**Mechanism of cancer metastasis**. **A**. Cancer cells produce proteases to destroy the surrounding matrix, and produce proteins to create new vessels. In cancer tissues, there are areas where the oxygen supply is poor, which induces hypoxia. Hypoxic cancer cells lose their adhesion characteristics and have enhanced capacity for migration. **B**. (1) Polyamines synthesized by cancer cells are transferred to cancer cells under hypoxic conditions that have increased capacity for polyamine uptake and decreased intracellular polyamine synthesis. The increase in polyamine concentration due to increased polyamine uptake decreases adhesion of cancer cells by decreasing adhesion molecule expression. (2) Polyamines are transferred to the blood cells. Increased polyamine uptake by immune cells results in decreased production of tumoricidal cytokines and the amount of adhesion molecules, and these eventually attenuate the cytotoxic activities of immune cells.

In cells exposed to chronic hypoxia, polyamine synthesis is decreased, while the ability to take up polyamines from the surroundings is increased [64,65]. Cells in a hypoxic environment have a resultant decrease in *de novo *polyamine synthesis and a concurrent increased capacity to take up polyamines from surrounding tissues, e.g. from cancer cells under normoxic conditions that are capable of producing abundant polyamines. We reported that cancer cells under hypoxia lose regulation of polyamine homeostasis and have increased polyamine uptake from surrounding tissues (Figure 2B, 1) [66]. The expression of the adhesion molecule CD44 is suppressed in response to hypoxia. Reduced CD44 expression is reported to promote cancer metastasis and invasion, allowing detachment of cancer cells from the primary tumor cluster and seems to contribute to the increased migration capacity of hypoxic HT-29 cells [67,68]. In conjunction with hypoxia, increases in extracellular spermine specifically augmented hypoxia-induced decreases in CD44 expression, and these decreases correlated well with increased migration of cancer cells (HT-29) in a dose-dependent manner [66]. In addition, several experiments indicated a possible role for polyamines in the invasive potential of cancer cells [53,55,69].

### 5-b. Role of polyamines in cancer cell transmigration to the circulation

Cancer invasion is the process in which cancer cells migrate through surrounding tissues and enter into a blood vessel, which enables cancer cells to be transported throughout the body and establish secondary tumors. Blood vessel entry requires that cancer cells not only have increased motility but also secrete enzymes that degrade the surrounding cells' extracellular matrix (ECM), which is composed of the interstitial matrix and basement membrane, and provides structural support to cells. Cancer cells produce various proteinases, such as serine proteinase, matrix metalloproteinases (MMPs), cathepsins, and plasminogen activator that degrade the ECM [70-72]. In addition, cancer cells have the ability to create new blood vessels in the tumor, i.e. angiogenesis, so that cancer cells can obtain supplies of blood and oxygen [73].

Increased polyamine synthesis appears to be accompanied by cancer invasiveness as ODC overexpression enhances the invasive characteristics of cancer cells [74]. In contrast, inhibition of polyamine synthesis by the ODC inhibitor DFMO attenuates the invasive characteristics of cancer cells [53,55,75], and supplementation with polyamine reverses the DFMO-induced decrease in invasive qualities [75]. The close correlation between increased polyamine synthesis and increased MMP synthesis has also been shown using DFMO, which caused decreases in cancer cell expression and concentrations of MMPs, such as matrilysin, meprin, and MMP-7 [76,77].

As mentioned above, increased polyamine synthesis is also accompanied by angiogenesis that is stimulated by cellular production of several factors, including vascular endothelial growth factor, which allow tumor tissues to grow and survive by obtaining sufficient blood supplies [78]. DFMO has been shown to exert its anti-tumor activity by inhibiting the proliferation of endothelial cells [79].

### 5-c. Possible role of polyamines on cell rooting and colonization at secondary tumor sites

Cancer cells that invade blood vessels and escape from immune system detection in circulation anchor to endothelial vasculature to establish new sites of growth. Upon vessel entry, cancer cells have access to abundant oxygen supplies that could enable cancer cells to restore their original activities such as increased gene expression that translates to enhanced enzymatic activities for polyamine synthesis, proteinase, and angiogenesis factors. Considering the results of our study, the expression of CD44 of normoxic cancer cells is higher than that of hypoxic cells [66], suggesting that the circulating cancer cells possibly recover their original adhesion characteristics. Once cancer cells anchor to the vessel wall of tissues and organs at secondary growth sites, they invade and rapidly grow because of their increased capacity to synthesize polyamines indispensable for cell growth and proteins that degrade the tissue matrix and create new vessels.

### 5-d. Polyamines help cancer cells escape immune system detection

Immune suppression, often observed in cancer patients, accelerates cancer spread. Various defects in cellular functions indicative of immune suppression have been reported, including attenuated adhesion properties of peripheral blood mononuclear cells (PBMCs) [80-82], impaired production of tumoricidal cytokines and chemokines [83-85], and decreased cytotoxic activity of killer cells, especially lymphokine activated killer (LAK) cells [86-89]. Several investigators have suggested that circulating factors that inhibit host immune activities are present in cancer patients [89-91]. The suppression of immune function in cancer patients can be restored following tumor eradication, further suggesting the presence of increased immunosuppressive substance(s) in cancer patients [83,84,89,91].

The increases in blood polyamine concentrations in cancer patients reflect increased polyamine concentrations in blood cells, mainly in red and white blood cells (Figure 2B, 2). The *in vitro *effects of polyamines on immune functions were first reported over 30 years ago [92]. However, later analysis revealed that the reported immunosuppressive effects are induced not by the direct effect of polyamines but by substances produced by the interaction between polyamines and serum amine oxidase, present exclusively in ruminants, making these results difficult to extend to humans, which lack this enzyme. Nonetheless, animal experiments have shown that polyamine deprivation prevents the development of tumor-induced immunosuppression [93].

The adhesion characteristics of immune cells are important for eliciting anti-tumor cytotoxic activity, because adhesion is crucial for immune cell recognition of tumor cells [94]. Due to decreased adhesion, immune cells fail to recognize cancer cells or exert tumoricidal activities. Such decreases in immune cell adhesion are observed not only in cancer patients but also in patients having non-cancerous lesions [82]. These findings suggest the possibility that common factor(s), not specifically produced in cancer patients, can induce immunosuppressive conditions. Polyamines are one such factor, because blood polyamine levels, namely levels in blood cells including immune cells, are often increased in patients with various diseases [36,95-97].

Immune cells also take up polyamines form their surroundings [98,99], and the increase in blood polyamine concentrations often observed in cancer patients as well as in patients with other diseases reflects the increased polyamine levels in leukocytes [36,100]. We have shown that increased concentrations of spermine or spermidine in cultured human PBMCs suppress adhesion without sacrificing cell viability and activity.

The time- and dose-dependent decrease in adhesion produced by polyamines was accompanied by decreases in the expression of lymphocyte function-associated antigen-1 (LFA-1), which consists of an integrin alpha L (CD11a) and beta 2 (CD18) chain [41]. Polyamines in particular decrease the number of cells expressing bright CD11a. Such suppression was exclusively observed for LFA-1 with most other adhesion molecules tested unaffected by polyamines. The suppression of LFA-1 expression by polyamines was further confirmed in human healthy volunteers with polyamines suppressing LFA-1 expression on PBMCs, regardless of the volunteer's age [41]. In addition to LFA-1 suppression by polyamines, the number of CD56 bright cells was decreased by polyamines *in vitro*, although the effect was not confirmed *in vivo*. LFA-1 and CD56 contribute to the induction of tumoricidal cell activities, especially lymphokine activated killer (LAK) activity [101,102]. LAK cells, which have tumoricidal activities against established (existing) tumors, are induced by co-culture with IL-2 [103,104]. In animal experiments, polyamine deprivation reversed the tumor inoculation-induced suppression of IL-2 production without decreasing the number of T lymphocytes [93]. In addition, polyamines (spermine and spermidine) inhibit the production of tumoricidal cytokines, such as tumor necrosis factor (TNF), and chemokines *in vitro*, while they do not inhibit production of transforming growth factor beta, which has immunosuppressive properties [105-107]. Conversely, in animal experiments, polyamine deprivation has been shown to enhance chemokine production, reverse tumor inoculation-induced inhibition of killer cell activity, and prevent tumor-induced immune suppression [108,109].

TNF is able to induce apoptotic cell death and to attack and destroy cancer cells [110], while LFA-1 and CD56, especially bright CD11a and bright CD56 cells, are required for the induction of LAK cell cytotoxic activity [111,112]. Polyamines suppress LAK cytotoxicity without decreasing cell viability and activity *in vitro*, and the changes in blood spermine levels are negatively associated with changes in LAK cytotoxicity in cancer patients [42].

## 6. Sources of polyamines other than cancer cells

Food is an important source of polyamines. Polyamines in the intestinal lumen are absorbed quickly and distributed to all organs and tissues [29,39,40]. Moreover, continuous intake of polyamine-rich food gradually increases blood polyamine levels [30,31]. Therefore, the restricted intake of food polyamine and inhibition of polyamine synthesis by microbiota in the intestine with or without inhibitor-induced inhibition of polyamine synthesis is reported to have favorable effects on cancer therapy [33,113-115].

Trauma, such as surgery, is itself considered to increase the risk of cancer spread through various mechanisms [116-118]. Blood concentration and urinary excretion of polyamines are known to increase after surgery, although the origin of this increase is not well established [97,119]. Our previous study showed that increases in blood polyamine levels are inversely associated with anti-tumor LAK cytotoxicities in patients who have undergone surgery [42]. In addition to mechanisms previously postulated for post-traumatic cancer spread, post-operative increases in polyamines may be another factor that accelerates tumor growth.

## Conclusion

As polyamines are essential for cell growth, one of the mechanisms by which polyamines accelerate tumor growth is through the increased availability of this indispensable growth factor. In addition, polyamines seem to accelerate tumor invasion and metastasis not only by suppressing immune system activity against established (already existing) tumors but also by enhancing the ability of invasive and metastatic capability of cancer cells. When considering the mechanism by which polyamines elicit their biological activities on immune and cancer cell functions, inhibition of polyamine uptake by cells seems to be an important target for polyamine-based cancer therapy particularly because inhibition of polyamine synthesis alone failed to produce a favorable effect on cancer treatments in several clinical trials. In addition to inhibiting polyamine synthesis and supply, inhibition of polyamine uptake via the polyamine transporter may have beneficial effects [120,121].

## List of abbreviations

APAO: N^1^-acetylpolyamine oxidase; DFMO: D, L-α-difluoromethylornithine; ECM: extracellular matrix; HIF-1: hypoxia inducible factor-1; LAK: lymphokine activated killer; LFA-1: lymphocyte function-associated antigen-1; MGBG: methylglyoxal bis-(guanylhydrazone); MMPs: matrix metalloproteinases; ODC: ornithine decarboxylase; PBMCs: peripheral blood mononuclear cells; SAM: S-Adenosylmethionine; SSAT: spermidine/spermine N1-acetyltransferase; TNF: tumor necrosis factor.

## Competing interests

The authors declare that they have no competing interests.

## Authors' contributions

KS contributed solely to the writing and submission of this work.
